# Clinical consequences of head and neck free-flap reconstructions in the DM population

**DOI:** 10.1038/s41598-021-85410-3

**Published:** 2021-03-16

**Authors:** Ting-Han Chiu, Chung-Kan Tsao, Sheng-Nan Chang, Jou-Wei Lin, Juey-Jen Hwang

**Affiliations:** 1 Department of Surgery, Chang Gung Memorial Hospital and Chang Gung University College of Medicine, Taoyuan, Taiwan; 2 Department of Plastic and Reconstructive Surgery, Chang Gung Memorial Hospital and Chang Gung University College of Medicine, Taoyuan, Taiwan; 3 Division of Cardiology, Department of Internal Medicine, National Taiwan University Hospital Yun-Lin Branch, Dou-Liu City, Taiwan; 4 Division of Cardiology, Department of Internal Medicine, National Taiwan University Hospital, Taipei, Taiwan

**Keywords:** Cardiology, Health care, Medical research, Risk factors

## Abstract

Diabetes mellitus (DM) is a common comorbidity and risk factor for postoperative complications in head and neck (H&N) microsurgical reconstructions. Our study focused on the association between DM and individual complications regarding both surgical and medical aspects. A meta-analysis of English-language articles comparing a series of complications between DM and non-DM H&N free-flap recipients was performed by comprehensive meta-analysis (CMA). Twenty-seven articles presented 14,233 H&N free-flap reconstructions, and a subset of 2329 analyses including diabetic cases was included for final analysis. Total postoperative (RR = 1.194, *p* < 0.001; OR = 1.506, *p* = 0.030) and surgical (RR = 1.550, *p* = 0.001; OR = 3.362, *p* < 0.001) complications were increased in DM subjects. Free-flap failure/necrosis (RR = 1.577, *p* = 0.001; OR = 1.999, *p* = 0.001) and surgical site infections (OR = 2.414, *p* < 0.001) were also increased in diabetic recipients. However, return to the operating room, dehiscence, fistulas, plate exposures, readmissions, and mortalities were not increased in DM patients. DM increased various complications in H&N free-flap reconstructions. Surgical indications should be cautiously evaluated, and aggressive treatments should be implemented for high-risk recipients.

## Introduction

Free-flap reconstruction following head and neck (H&N) tumor ablation has become a routine practice with advancements in microsurgical techniques^1^. However, daunting postoperative complications after free-flap reconstruction, such as deprivation of eating, speaking, and breathing functions, have been reported^2^. Numerous practices have been introduced to improve surgical outcomes after free-flap reconstruction, especially risk factor evaluation^3^. Among those factors associated with postoperative complications, diabetes mellitus (DM) is a very important risk factor in clinical practice. However, there have been differing reports about free-flap success rates, surgical site infections, and surgical outcomes in DM patients in previous studies^4–7^.

A lack of persuasive and clinical evidence impedes determining whether DM is contributory to complications following major microsurgical procedures. As tissues left after H&N tumor resections are often scarce, free-flap reconstruction is frequently used for instant coverage and functional restorations. Serious postoperative complications after free-flap reconstruction not only psychologically devastate patients with physical appearance or loss of basic function but also may delay crucial radiotherapy^4,5^. In those cases, the quality of life after the operation is not satisfactory due to repeated readmissions or revision operations^8–10^. Therefore, recognizing the risk of DM in association with free-flap reconstruction and the response to corresponding treatments before free-flap reconstruction are very important in real-world practice. To define the possible effects of DM in association with free-flap use for H&N reconstruction, we performed this meta-analysis to examine these associated complications in more detail.

## Patients and methods

### Inclusion criteria

All published original studies of either randomized control trials or retrospective cohorts mentioning DM and any certain complications following H&N free-flap reconstructions were included. Articles by the same authors or based on the same study population should be verified as separate studies or designated to analyze a different complication before being included.

### Search strategy

A search of electronic databases, namely PubMed Central, Embase, MEDLINE, and the Cochrane Library, from January 2005 to April 2020 was conducted; the search terms were “DM”, “H&N,” AND “free flaps.” This was further supplemented with cross-referencing the bibliographies from the papers identified by the search or other relevant articles. Only full-text manuscripts in the English language were considered for inclusion. For studies with overlapping periods of the same targeted population, the one with the largest cohort was retained, while additional consideration for inclusion was made if the overlapping paper mentioned a specific complication that was not previously covered. Articles without available documentation of the diabetic or nondiabetic case numbers and respective complication rates or without an odds ratio comparing risks in diabetic to those in nondiabetic patients were further excluded.

### Data extraction

After selecting the relevant literature, the primary data collection was performed and further reviewed by a second author to ensure accuracy. The data collected were as follows: definition of the reconstructed regions, study population and years, numbers of total patients and patients with DM, and rates of complications in both patients with and without DM. Various complications (surgical and medical), surgical complications, return to the operating room, free-flap failure/necrosis, surgical site infections, dehiscence, hematomas, coagulation-related complications (e.g., hematoma, bleeding, requiring transfusion or thrombosis), fistulas, plate exposures, other complications, readmissions, and mortality were included in the complication analysis.

### Statistical analysis

The meta-analysis was conducted using Comprehensive Meta-Analysis (CMA) 2.0 Software, [Biostat, New Jersey, USA], using the inverse variance method for pooled relative risk. The dichotomous data were summarized using relative risk (RR) or odds ratio (OR) separately and with 95% confidence intervals (95% CIs). Statistical heterogeneity tests of Cochran's Q-value, *I*^2^, and Tau^2^ were performed to determine whether a fixed or random effect model was adopted.

## Results

### Process outcomes

**Figure 1:**
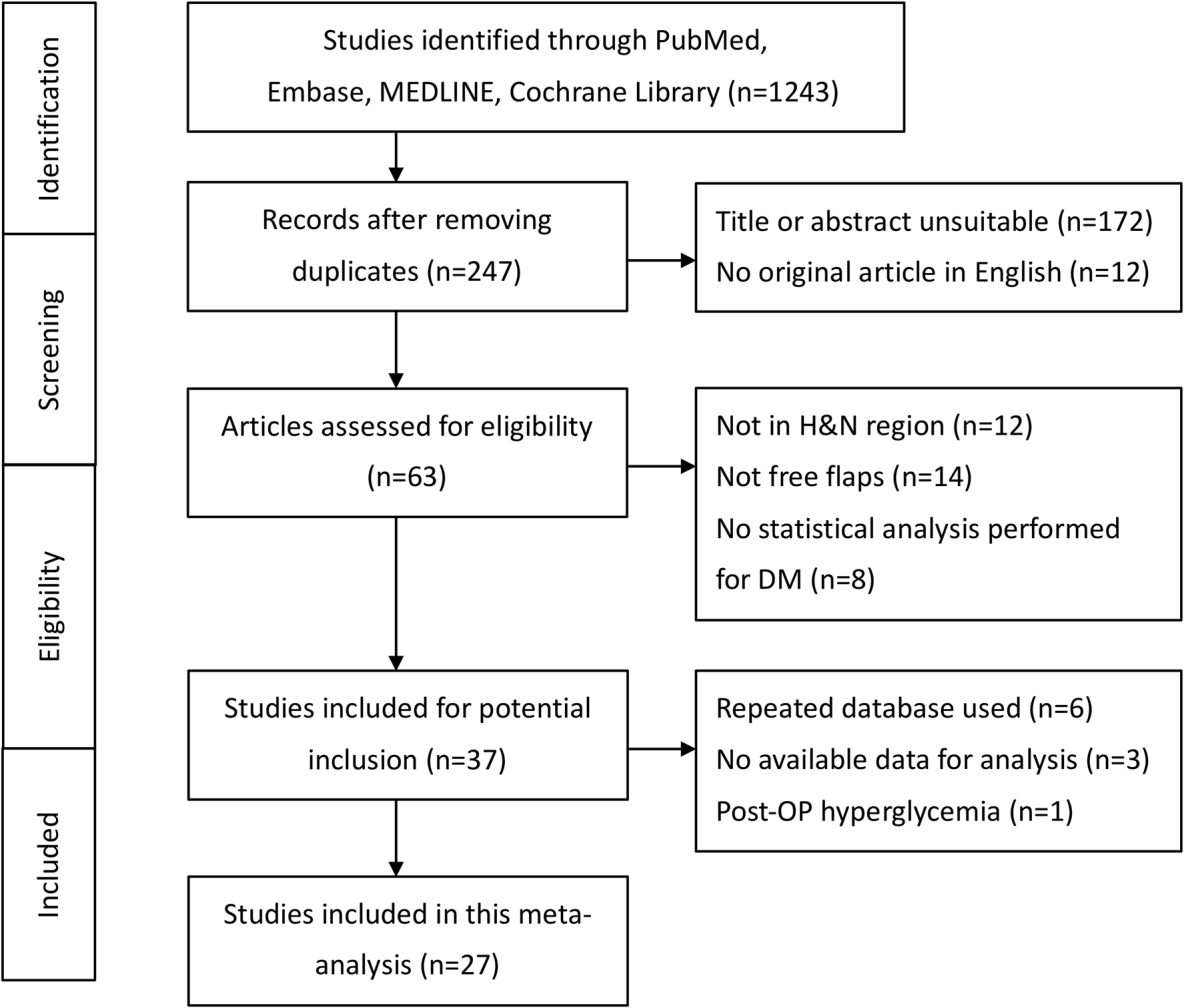
The flow diagram shows the process of enrolling studies for the meta-analysis.

**Figure 2:**
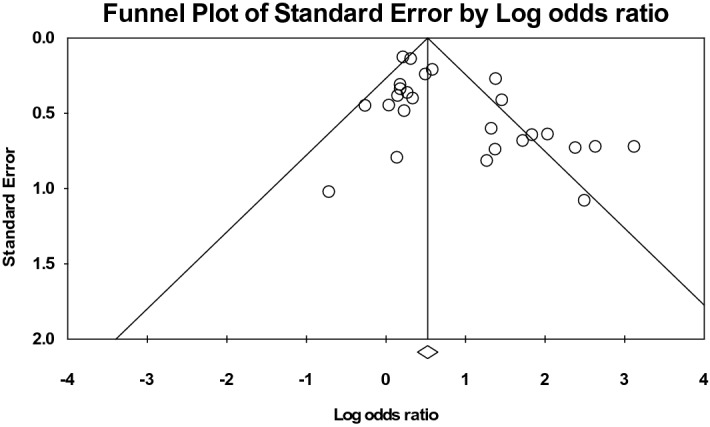
The funnel plot of all studies included.

The search strategy identified 63 eligible studies, and 27 studies were ultimately included in the meta-analysis^5,11–37^. The selection process is demonstrated in the flow diagram (Fig. 1), and the study characteristics are presented in Table 1. The funnel plot of all studies included is presented in Fig. 2.

**Table 1:** Patient Characteristics of the studies included in the meta-analysis (n = 27).

References	Study Design	Database	Flap definition	No. of patients	No. of DM patients	Analyzed complication
Bozikiv^11^	Retrospective cohort	University Medical Centre Ljubljana, Slovenia 1989–1999	H&N free flaps following tumor ablations	162	12	Surgical complications (RR), flap failure/necrosis (RR)
Valentini^12^	Retrospective cohort	Polyclinic "Umberto I," University of Rome, Italy 2001–2004	H&N free flaps	118	8	Return to OR (RR), hematoma (RR)
Naura^13^	Retrospective cohort	Cleveland Clinic, United States 6 years	H&N free flaps	300	28	Surgical complications (RR), return to OR (RR), flap failure/necrosis (RR), surgical site infection (RR), dehiscence (RR), hematoma (RR), fistula (RR), plate exposure (RR)
Kao^14^	Retrospective cohort	Chang Gung Memorial Hospital, Taiwan 2000–2008	H&N free flaps after cancer ablations	62	16	Surgical complications (RR), return to OR (RR)
Bianchi^15^	Retrospective cohort	University of Parma, Italy 2000–2007	H&N free flaps	352	24	Total complications (RR, OR)
Joo^16^	Retrospective cohort	Catholic University of Korea, Korea 1993–2009	H&N free flaps after ablative surgery	237	27	Flap failure/necrosis (RR, OR)
Lee^17^	Retrospective cohort	University of Jena, Germany 2008–2009	H&N free flaps	81	7	Surgical complications (RR, OR)
le Nobel^18^	Retrospective cohort	Sunnybrook Health Science Center, Canada 2003–2010	H&N free flaps	289	35	Total complications (RR, OR)
Vandersteen^19^	Retrospective cohort	Institut Universitaire de la Face et du Cou, France 2000–2010	H&N free flaps	423	35	Total complications (RR), surgical complications (RR), surgical site infection (RR), hematoma (RR), fistula (RR), mortality (RR)
Riva^20^	Retrospective cohort	Kaohsiung Chang Gung Memorial Hospital, Taiwan 1996–2008	H&N free flaps after ablations	1233	189	Flap failure/necrosis (RR)
Avery^21^	Retrospective cohort	University Hospitals of Leicester, United Kingdom 1996–2012	Free pectoralis major flaps for maxillofacial regions	100	16	Flap failure/necrosis (OR)
Liu^22^	Retrospective cohort	Shanghai Ninth People’s Hospital, China 2003–2013	H&N free flaps for oral cancers	309	105	Surgical complications (RR, OR), return to OR (RR), flap failure/necrosis (RR), surgical site infection (RR), dehiscence (RR), hematoma (RR), thrombosis (RR)
Mitchell^23^	Retrospective cohort	University of Washington Medical Center or Harborview Medical Center, Unites States 2006–2013	H&N free flaps (clean-contaminated wounds)	427	40	Surgical complications (OR)
Lo^24^	Retrospective cohort	Cathay General Hospital, Taiwan 2010–2014	H&N free flaps after cancer ablations	158	32	Total complications (RR)
Ishimaru^25^	Retrospective cohort	National Inpatient Database, Japan 2010–2013	H&N free flaps after tumor resections	2846	737	Flap failure/necrosis (RR, OR)
Eder-Czembirek^26^	Retrospective cohort	Vienna General Hospital, Austria 2004–2011	Free-flap reconstructions for oral squamous cell carcinoma	85	9	Surgical site infection (RR, OR)
Zhou^27^	Retrospective cohort	Peking University School and Hospital of Stomatology, China 2013–2016	H&N free flaps	881	65	Flap failure/necrosis (RR)
Yao^28^	Retrospective cohort	University Health Network in Toronto, Canada 1997–2014	Free-flap reconstructions for oral squamous cell carcinoma	365	39	Surgical site infection (RR), plate exposure (RR)
Joo^29^	Retrospective cohort	Catholic University of Korea, Korea 1993–2014	Free-flap reconstructions for H&N squamous cell carcinoma	259	40	Surgical site infection (OR)
Khan^30^	Retrospective cohort	Mount Sinai Medical Center, United States 2007–2014	H&N free flaps	415	46	Surgical site infection (OR)
Brady^5^	Retrospective cohort	National Surgical Quality Improvement Program, United States 2005–2014	H&N free flaps	2187	256	Total complications (RR), surgical complications (RR), return to OR (RR), flap failure/necrosis (RR), surgical site infection (RR), dehiscence (RR), bleeding (RR), other complications (RR), readmission (RR), mortality (RR)
Bollig^31^	Retrospective cohort	Missouri Hospital, United States 2009–2015	H&N free flaps	203	91	Surgical complications (RR), surgical site infection (RR), venous thrombosis (RR)
Eskander^32^	Retrospective cohort	Ohio State University, United States 2006–2012	H&N free flaps	515	84	Total complications (RR, OR), surgical site infection (RR, OR), dehiscence (RR), other complications (RR)
Rudolph^33^	Retrospective cohort	Wake Forest Baptist Medical Center, United States 2008–2016	Cross-paramedian forehead flaps for nasal reconstructions	53	9	Surgical complications (OR)
Eskander^35^	Retrospective cohort	Ohio State University, United States 2006–2012	H&N free flaps	515	84	Readmission (RR)
Crawley^36^	Retrospective cohort	Thomas Jefferson University, United States 2006–2017	H&N free flaps	889	128	Flap failure/necrosis (RR)
Lin^37^	Retrospective cohort	Kaohsiung Chang Gung Memorial Hospital, Taiwan 2008–2017	H&N free anterolateral thigh flaps	1284	251	Surgical complications (RR)

### Analysis of complications

#### Total complications

**Figure 3:**
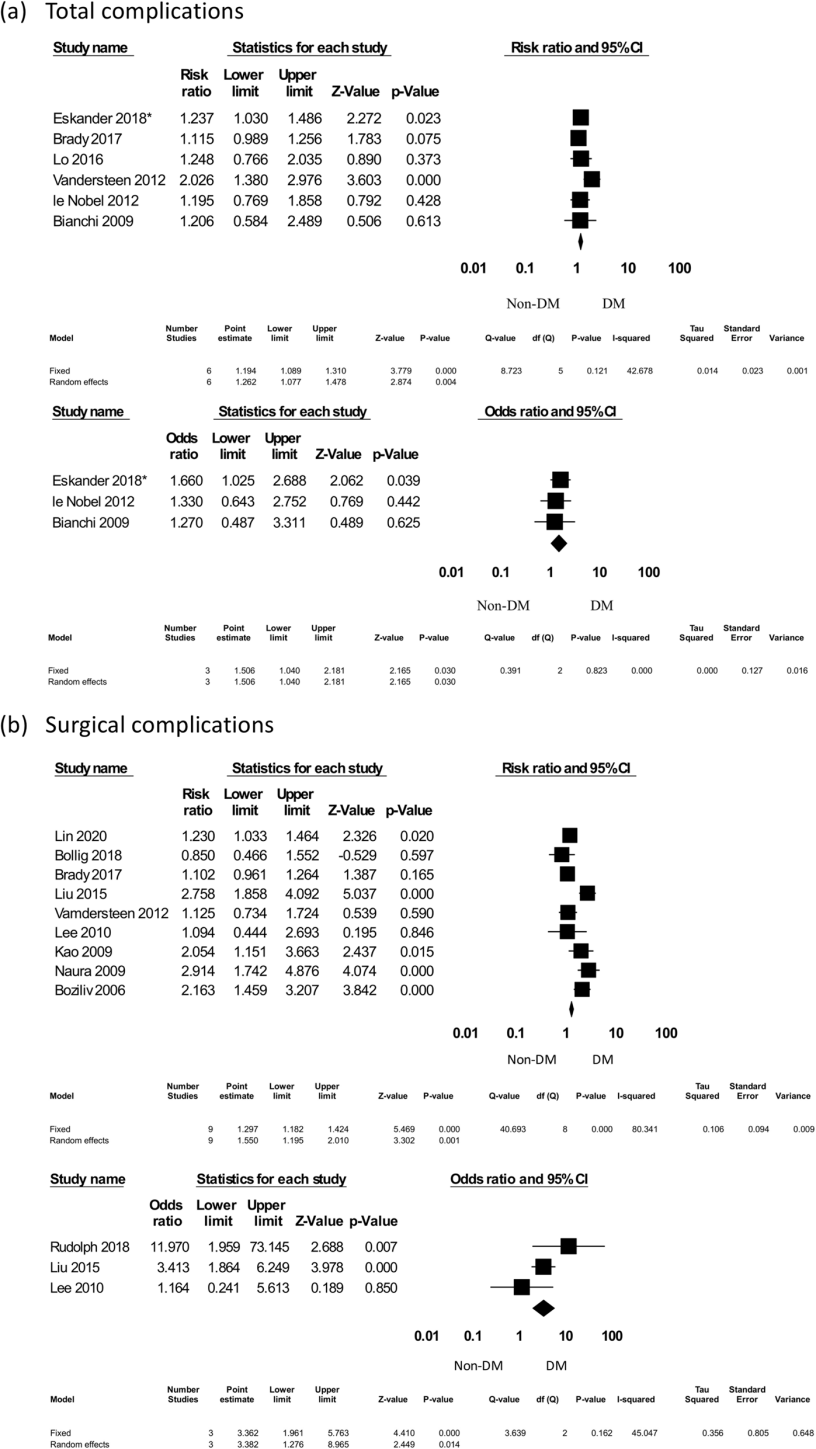
The postoperative complications forest plot: total and surgical complications. *Eskander^32^.

A total of 6 studies were included for overall postoperative, surgical and other complications. A total of 3924 patients were enrolled, 466 of whom had DM. The pooled results were 6 studies with RR and 3 studies with OR. After multivariable analysis, DM significantly increased the total complication rate (RR = 1.194, 95% CI 1.089–1.310, *p* < 0.001 and OR = 1.506, 95% CI 1.040–2.181, *p* = 0.030) (Fig. 3).

#### Total and individual surgical complications

##### Total surgical complications

Ten studies that evaluated total surgical complications were identified; a total of 5064 patients were enrolled, 744 of whom were DM patients. The pooled results include 9 studies with RR and 3 studies with OR. The multivariate analysis results showed that DM significantly increased surgical complication rates (RR = 1.550, 95% CI 1.195–2.010, *p* = 0.001 and OR = 3.362, 95% CI 1.961–5.763, *p* < 0.001) (Fig. 3).

##### Return to the operating room

**Figure 4:**
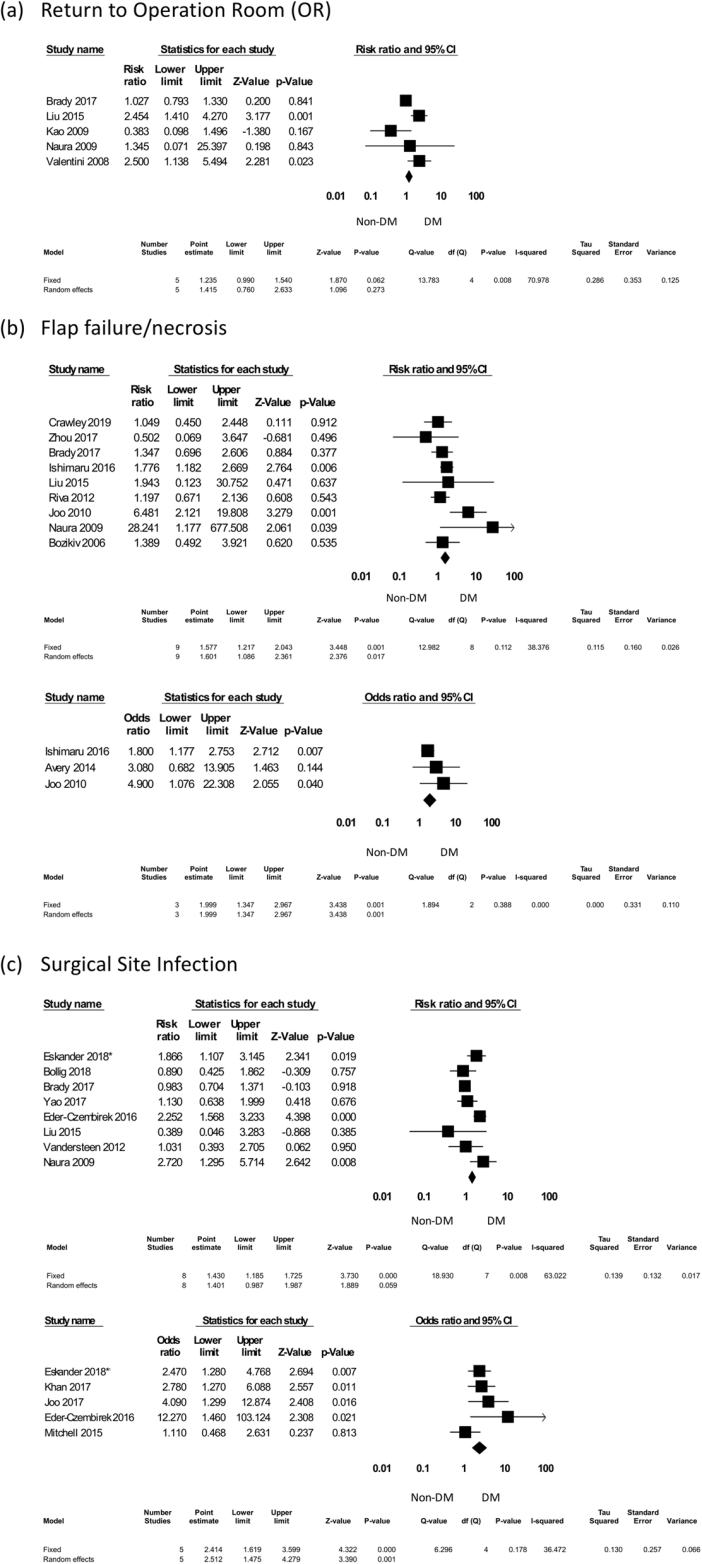
The individual surgical complications forest plot, part I: return to the operating room, flap failure/necrosis, and surgical site infection. *Eskander^32^.

Five studies evaluating the rates of “return to the operating room” were included, with 413 DM patients among 2976 total subjects. All complication rates were expressed as RR. The pooled results showed that DM did not significantly increase the rate of “return to the operating room” (RR = 1.415, 95% CI 0.760–2.633, *p* = 0.273) (Fig. 4).

##### Flap failure/necrosis

Ten studies that evaluated flap failure or necrosis were included, with 9144 total patients and 1550 DM patients. The pooled results included 9 studies with RR and 3 studies with OR. The multivariate analysis results showed that DM significantly increased flap failure or necrosis (RR = 1.577, 95% CI 1.217–2.043, *p* = 0.001 and OR = 1.999, 95% CI 1.347–2.967, *p* = 0.001) (Fig. 4).

##### Surgical site infections

A total of 11 studies evaluated surgical site infections, with 5488 patients enrolled; 773 of them had DM. The pooled results included 8 studies with RR. There was a trend of increasing surgical site infections in the DM group, nearly reaching statistical significance (RR = 1.401, 95% CI 0.987–1.987, *p* = 0.059). After pooling the results from the 5 studies that reported OR, DM significantly increased surgical site infections (OR = 2.414, 95% CI 1.619–3.599, *p* < 0.001) (Fig. 4).

##### Dehiscence

**Figure 5:**
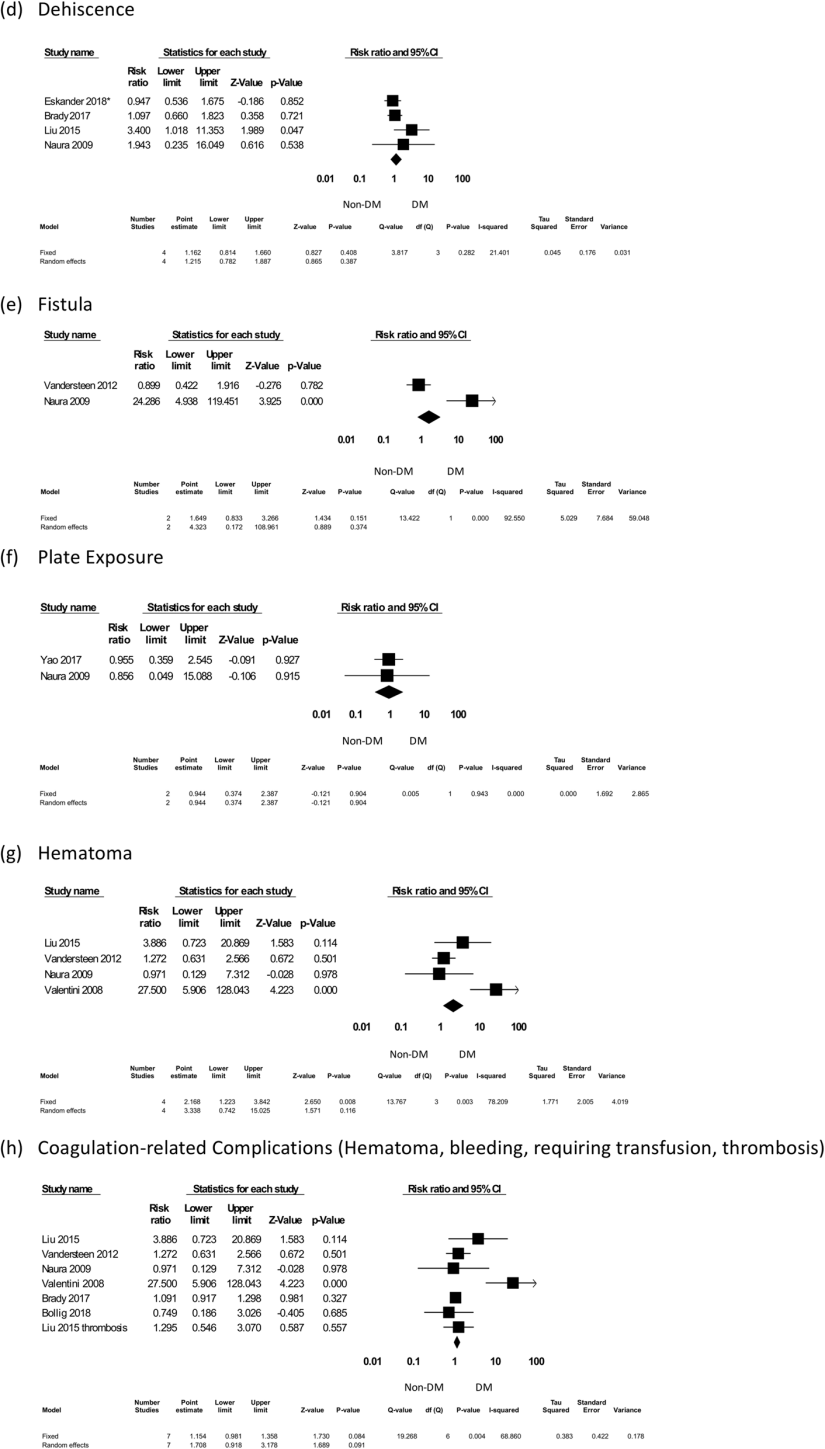
The individual surgical complications forest plot, part II: dehiscence, fistula, plate exposures, hematoma, and coagulation-related complications. *Eskander^32^.

Four studies were included to evaluate dehiscence rates, with 473 DM patients among 3311 total subjects. All complication rates were expressed as RR. The pooled results showed that DM did not significantly increase dehiscence rates (RR = 1.162, 95% CI 0.814–1.660, *p* = 0.408) (Fig. 5).

##### Fistula

Two studies were included to evaluate fistula formation after surgery, with 63 DM patients among 723 subjects. All complication rates were expressed as RR. The pooled results showed that DM did not significantly increase fistula formation after the operation (RR = 4.323, 95% CI 0.172–108.961, *p* = 0.374) (Fig. 5).

##### Plate exposure

Two studies were included for the evaluation of plate exposures, with 67 DM patients among 665 subjects. All complication rates were expressed as RR. The pooled results showed that DM did not increase plate exposure rates (RR = 0.944, 95% CI 0.374–2.387, *p* = 0.904) (Fig. 5).

##### Hematoma

Four studies were included for the evaluation of hematoma, with 176 DM patients among 1150 subjects. All complication rates were expressed as RR. The pooled results showed that DM did not significantly increase the rates of hematoma (RR = 3.338, 95% CI 0.742–15.025, *p* = 0.116) (Fig. 5).

##### Coagulation-related complications

Regarding coagulation-related complications (e.g., hematoma, bleeding, required transfusion, and thrombosis), seven studies were included in the meta-analysis. The complication rates were expressed as RR; there were a total of 3849 patients, 562 of whom were DM patients. The pooled results showed that there was a trend of increasing coagulation-related complications in the DM group (RR = 1.708, 95% CI 0.918–3.178, *p* = 0.091) (Fig. 5).

#### Quality aspects

##### Readmissions

**Figure 6:**
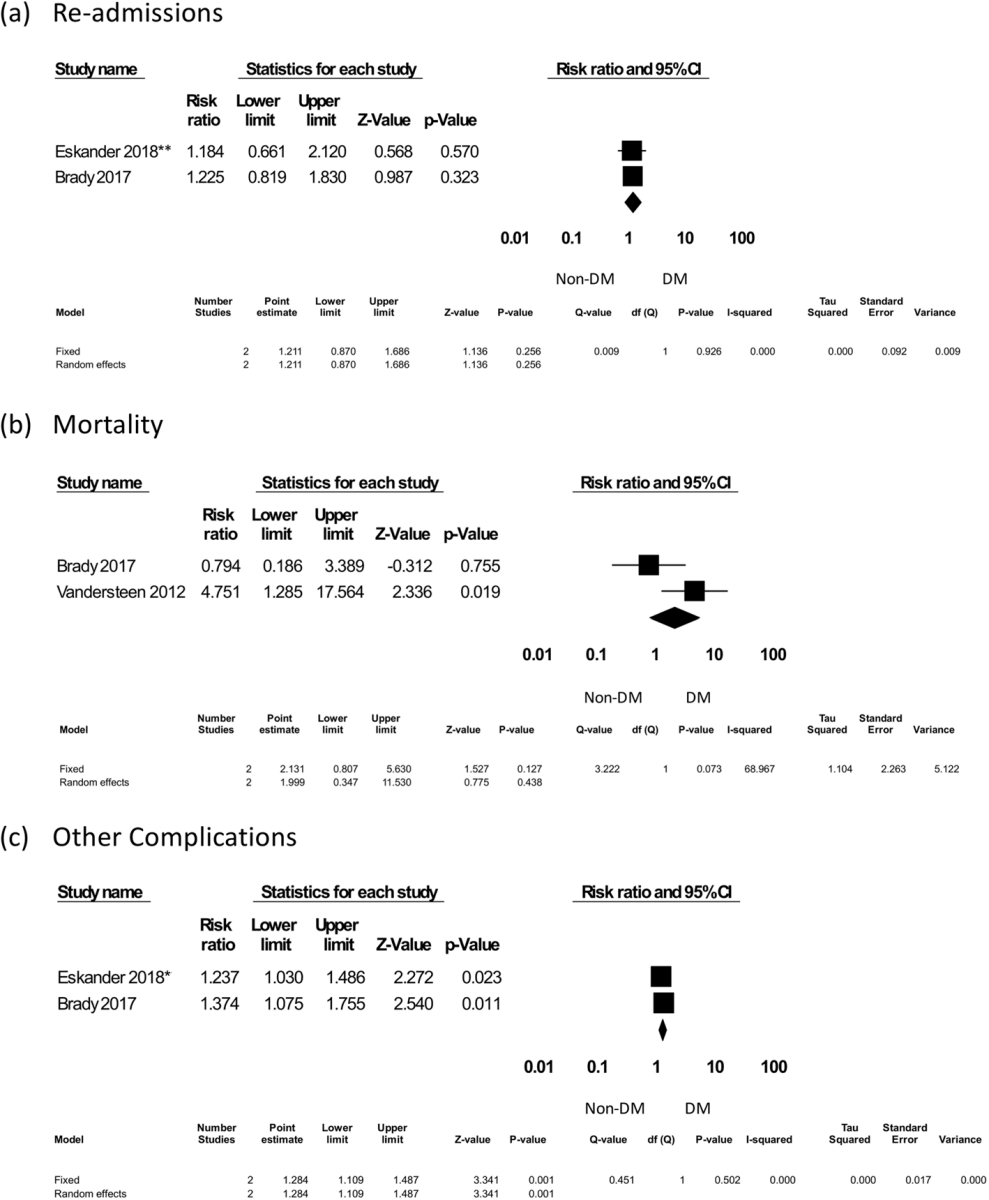
Quality aspects: readmissions, mortality, and other complications. *Eskander^32^, **Eskander^35^. Other complications include myocardial infarction, cerebrovascular event, deep vein thrombosis, pulmonary embolism, pneumonia, urinary tract infection, and septic shock.

Two studies were included to evaluate the rates of readmission, with 340 DM patients among 2702 subjects. All complication rates were expressed as RR. The pooled results showed that DM did not significantly increase readmission rates (RR = 1.211, 95% CI 0.870–1.686, *p* = 0.256) (Fig. 6).

##### Mortality

Two studies were included to evaluate mortality, with 291 DM patients among 2610 subjects. All complication rates were expressed as RR. The pooled results showed that DM did not significantly increase mortality rates (RR = 1.999, 95% CI 0.347–11.530, *p* = 0.438) (Fig. 6).

##### Other complications

Two studies were included in the evaluation of other complications (e.g., myocardial infarction, cerebrovascular event, deep vein thrombosis, pulmonary embolism, pneumonia, urinary tract infection, and septic shock); a total of 2702 patients were enrolled, including 340 DM patients. All complication rates were expressed as RR. The pooled results showed that DM significantly increased the risk of other complications (RR = 1.284, 95% CI 1.109–1.487, *p* = 0.001) (Fig. 6).

## Discussion

DM has been investigated for potentially increasing the risk of postoperative complications, such as vascular occlusions and thrombosis, that jeopardize free flap survival and result in immune disturbances associated with poor wound healing^8–10^. However, retrospective cohort studies from different institutions and even different time periods within the same database could end up with contradicting conclusions^4,5^.

Previous meta-analyses exploring the association between DM and H&N free-flap reconstructions had a few limitations. In 2015, Rosado et al. noted that DM was significantly related to more surgical complications following free-flap reconstructions in the H&N region^38^. Consisting of merely five studies, this analysis seemed relatively short of conclusiveness^38^. Further investigations were performed by Cupato et al.^39^; these authors gathered 16 studies for meta-analysis. They concluded that DM significantly increased flap failures and local region complications^39^. This complication analysis was performed to include all major H&N surgeries instead of limiting the analysis to free-flap reconstructions^39^. They also pooled different complications under the term “locoregional”^39^. None of these meta-analyses was explicitly designed to analyze individual complications. Additionally, neither one considered crucial complications nor quality indicators in their studies. For surgeons, a better understanding of which complication was at higher risk might be more helpful and provide insights into routine or pragmatic formulations in clinical practice.

In this study, a total of 27 studies regarding H&N free-flap reconstructions in patients with DM were included in a meta-analysis in response to preceding studies with different opinions about free-flap failure or abnormal healing of the anastomoses in DM subjects^5,12,40,41^. Some studies concluded that patients with DMS were at increased risk and others did not^5,12,40,41^. Overall, we found that DM was associated with increasing total complications (surgical and other clinical sequelae), return to the operating room, and free-flap failure after the operation (Figs. 3 and 4). The underlying mechanisms might be due to the high prevalence of peripheral vascular diseases in DM patients^8^. Peripheral vascular diseases can result in fragile vessel conditions and precipitate free-flap failure. Currently, vascular mapping for evaluating vascular viability and anatomy by color Doppler, computed tomography (CT) angiography or magnetic resonance imaging (MRI) angiography might improve microsurgical outcomes^42^. Therefore, adopting angiography as part of the preoperative assessments might be beneficial and suggested for routine used in DM patients referred for free-flap operations.

DM has been demonstrated to alter the immune system with cytokine effects on local wound healing^10^. Higher infection rates in DM patients have been previously reported from various types of surgical procedures^6^. In our study, surgical site infection after free-flap reconstruction was significantly increased in DM patients (RR = 1.401, *p* = 0.059; OR = 2.414, *p* < 0.001) (Fig. 4). Therefore, hyperglycemia should be taken seriously and controlled cautiously to reduce surgical site infections in DM patients before and after surgery.

The ideal target for glycemic control before and after surgery is still a debated issue. In some studies, maintaining HbA1c less than 8.5% (69 mmol/mol) for the general population undergoing surgeries is highly suggested^43^. However, there is no consensus about whether DM patients planning to undergo free-flap reconstructions should maintain stricter glycemic control. Currently, intensive blood sugar control of less than 150 mg/dL before and after surgery might reduce the risk of surgical site infection^44^. Both the Society of Thoracic Surgeons (STS) and the American Association of Clinical Endocrinologists and American Diabetes Association (AACE/ADA) endorse a glycemic range between 140 and 180 mg/dL for postoperative patients^45^.

A series of analyses according to the infection-related complications were further performed. Interestingly, DM was not associated with dehiscence, fistulas, or plate exposure (Fig. 5). Early empirical antibiotic usage or switching, surgical intervention and return to the operating room might control infection and deter such serious and long-term complications. However, there has been no consensus regarding infection prophylaxis and management in patients with DM following microsurgical reconstructions^23,46^.

Pharmacologic prophylaxis with antiplatelets, anticoagulants, and volume expanders has been commonly prescribed after free flap transfers in DM patients due to their impaired microcirculation^47^. In this study, of the risk of hematoma was not increasing in diabetic flap recipients (Fig. 5). However, there was a trend of increasing total coagulation-related complications (e.g., hematoma, bleeding, required transfusion, and thrombosis) in the DM group (RR = 1.708, 95% CI 0.918–3.178, *p* = 0.091) (Fig. 5). Therefore, surgeons should be cautious in dose adjustment and coagulation monitoring while using antiplatelets or anticoagulants in DM patients after surgery^47^.

Regarding the quality aspects, we found that DM did not significantly increase readmission rates or mortality after free-flap reconstruction. However, other common clinical complications, including myocardial infarction, cerebrovascular event, deep vein thrombosis, pulmonary embolism, pneumonia, urinary tract infection, and septic shock, were noted to be increased after surgery in DM patients. It might be that those patients with H&N malignancies seemed to suffer from more comorbidities than the general population^48,49^. For instance, the high prevalence of tobacco and alcohol use in those patients imposes higher cardiovascular diseases, which could be exacerbated during surgery^50,51^. Additionally, blood transfusion during or after surgery might also aggravate heart failure, respiratory distress, and pneumonia^52^. Aside from cancer recurrence or metastasis, the most common causes of mortality after free-flap reconstruction were cardiac, pulmonary, and infectious etiologies^53^. Therefore, pre- or postoperative care should be more attentive for DM patients undergoing H&N reconstructions. A thorough examination of cardiopulmonary function and comprehensive comorbidity treatments should be implemented before the operation. In addition to surgical teams, different specialties might be consulted beforehand to avoid serious medical consequences.

## Conclusion

In summary, DM patients are prone to develop various complications after H&N free-flap reconstructions, and more aggressive strategies should be taken to ensure better outcomes. Our study results suggest practical ways for surgeons and oncologists to evaluate the risk of surgery in these patients. A patient-based and individual decision-making process should always be implemented and cautiously reviewed before free-flap reconstruction.
